# Responses of Labile Organic Nitrogen Fractions and Enzyme Activities in eroded Mollisols After 8-year Manure Amendment

**DOI:** 10.1038/s41598-018-32649-y

**Published:** 2018-09-21

**Authors:** Yi-min Chen, Xin Xu, Xiao-guang Jiao, Yue-yu Sui, Xiao-bing Liu, Jin-yuan Zhang, Ke Zhou, Jiu-ming Zhang

**Affiliations:** 1 Key Laboratory of Mollisols Agroecology, Northeast Institute of Geography and Agroecology, Chinese Academy of Sciences, 150081 Harbin, China; 2 College of Agricultural Resources and Environment, Heilongjiang University, 150080 Harbin, China; 3 Institute of Soil and Fertilizer and Environment Resources, Heilongjiang Academy of Agricultural Sciences, Harbin, 150086 China; 4 University of Chinese Academy of Sciences, 100049 Beijing, China

## Abstract

Soil erosion will cause a degradation in soil nitrogen supplying capacity (SNSC) and manure amendment is an effective way to restored eroded soils. Both labile fractions of soil organic N (SON) and N transformation enzymes are indicators for SNSC, but the effect of manure amendments on labile SON fractions and the relationship between labile SON fractions and enzyme activities remains unclear. In this study, five degrees of erosion were simulated in Mollisols (removal of 0, 5, 10, 20 and 30 cm of topsoil) to analyse the changes in labile SON fractions and nitrogen transformation enzyme activities after 8-year manure amendment. We found that soil total N (TN), labile SON fractions and enzyme activities all increased after manure amendments. The largest labile SON fraction was particle organic nitrogen (POM-N) and the second was light fraction organic nitrogen (LFOM-N), which accounted >60% for TN in total. Correlation analysis showed that both urease and protease activities were significantly correlated with POM-N, LFOM-N, microbial biomass N and dissolvable organic N, indicating that both urease and protease activities can be used to predict labile SON pools and enzyme activities worked similarly in indicating SNSC with labile SON fractions. Altogether, 8-year manure amendment could recover SNSC of lightly eroded Mollisols to natural levels, i.e. erosion depths at 5 cm and 10 cm; however, it is not able to recover SNSC in Mollisols suffering severe erosion.

## Introduction

Nitrogen (N) is a primary element which is vitally important for life^1^. Both plant growth and crop production largely depend on the soil N supplying capacity (SNSC)^2^. In soil, most N exist in the form of soil organic nitrogen (SON), and over 90% of the N in the surface layer of most soils is organically bound^3^. N mineralized from SON contributes a lot to N needed by crops and may supply more than 50% of total crop N uptake^4,5^. Considering the importance of SON in N supplying, it is urgent to gain a further understanding of SON, such as the labile fractions of SON.

Most mineralized N is considered to derive from the labile SON fractions, namely particulate organic nitrogen (POM-N), light fraction organic nitrogen (LFOM-N), microbial biomass nitrogen (MBN), and dissolvable organic nitrogen (DON)^6^, due to their rapid transformation rates. Labile organic N fractions are important indicators for the SNSC^7^, as they are responsive to management changes and seasonal N availability^8^. MBN is considered as both a source and a pool of mineralized N^9,10^ and is thought to be the most labile SON fraction due to its metabolic function^11^. DON, which is derived from the degradation of organic residues and SON^12,13^, is readily metabolized within hours to days^14^ and shows a strong positive correlation with mineralized N in soil^15^. The LFOM-N and POM-N fractions are major N source for microbes^16^, as containing partially decomposed residues and some microbial by-products. POM-N is always considered as an indicator of SNSC in view of their key role in N-mineralization^17^ and N-availability to crops^8^. Thus, investigating labile SON fractions should help to gain a more comprehensive understanding of SNSC and the stability of soil N pool.

N transformation was regulated by microorganisms and was directly catalysed by the enzymes secreted by microbes^18^. The enzyme urease catalyses the hydrolysis of urea to CO_2_ and NH_3_ with the concomitant rise in soil pH. This in turn, results in a rapid N loss to the atmosphere through NH_3_ volatilization. With respect to this, urease activity plays an important role in the regulation of N supply^19^. Proteins represent by far the largest pool of organic N in soil^20,21^. Proteolysis catalysed by protease is an important process with regard to N-cycling because it is considered to be a rate-limiting step during N mineralization in soils due to the much slower primary phase of protease activities during N mineralization compared with amino acids mineralization^20,22,23^. Both N transformation enzyme activities and labile organic nitrogen fractions can be used as indicators for SNSC, however, the relationships between them remains unclear. It is imperative to determine whether they function similarly in indicating SNSC.

Soil erosion, an irreversible process caused by water, wind, and ice^24^, will cause significant degradation of soil properties such as nutrient availability, texture, structure, and water-holding capacity^25,26^. Previous investigations indicate that SOM declines with increased severity of soil erosion^27–29^, and as a consequence, the SON will also decline. Therefore, the loss of SON caused by soil erosion would negatively affect the SNSC, resulting in losses in crop yield. To combat soil erosion, organic amendments have frequently been proposed to reduce soil erodibility, e.g. by protecting aggregate surfaces against rainfall impact and providing substrates for microbial transformation into compounds acting as glue between soil particles^22,30–34^.

Chinese Mollisols are one of the most fertile soils in the world, but have suffered from severe erosion in recent years. Soil erosion and associated yield suppression have been a serious problem threatening agriculture sustainability in this region for decades^35,36^. Manure has been used to restore eroded Mollisols and soil quality of eroded Mollisols has been improved largely^28,37–39^. Moreover, the application of cattle manure can even recover the yields on eroded Mollisols to normal levels^40^. However, little information on how manure amendment affects labile organic nitrogen fractions and enzyme activities in eroded Mollisols is obtained.

The objectives of this study were to determine i) how labile SON fractions were affected in eroded Mollisols of different erosion depths after maunre amentment, ii) the relationships between nitrogen transformation enzymes activities and labile SON fractions and iii) the restorative effects of manure on Mollisols of different erosion degrees.

## Results

### Soil total nitrogen

**Figure 1:**
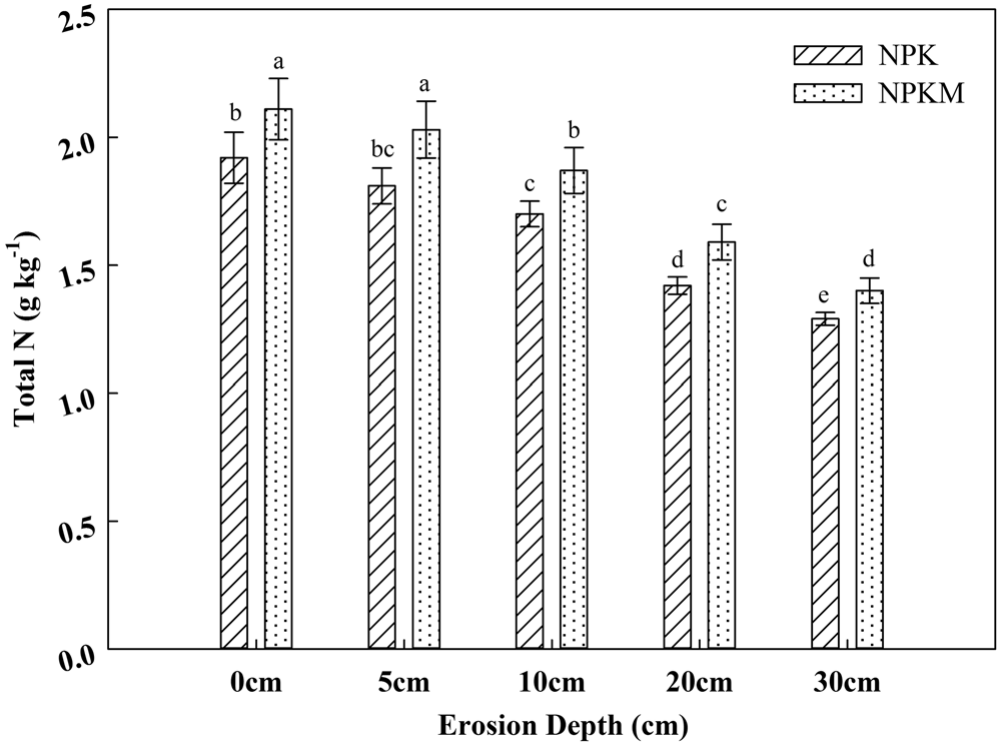
Total N in Mollisols at different depths of erosion. NPK in the legend represents the treatment with chemical fertilizers alone, and NPKM represents the treatment with chemical fertilizers plus manure amendment. Different letters above bars indicate that means were significantly different at the level of 0.05. Bars represent standard errors (n = 3).

The changes in TN was similar in both NPK and NPKM treatments, which decreased with the increasing erosion depth. From an erosion depth of 10 cm, TN decreased significantly (*p* < *0*.*05*). Of the five depths of soil erosion, the highest TN contents were in 0 cm, which were 2.11 g kg^−1^ in the NPKM treatment and 1.91 g kg^−1^ in the NPK treatment, whereas the lowest TN contents were in the 30 cm depth, which were 1.40 g kg^−1^ in the NPKM treatment and 1.29 g kg^−1^ in the NPK treatment (Fig. 1). Compared with the NPK treatment, TN increased by 8.53% ~12.15% in the NPKM treatment. The increasing rates also decreased with the increasing erosion depths from 5 cm to 30 cm. Manure amendment promoted the accumulation of TN in eroded Mollisols.

### The contents of soil labile organic nitrogen fractions

Similar to the results for TN, POM-N, LOFM-N and MBN all decreased with increasing erosion depth (Table 1). For a specific erosion depth, manure amendment could significantly increase the contents of POM-N and LFOM-N, with increasing rates 18.94%~32.77% for POM-N and 15.49%~18.52% for LFOM-N. MBC also increased after manure amendment with increasing rates 21.31%~44.68%, but in erosion depths 20 cm and 30 cm, no significant difference was detected. The increasing rates of POM-N, LFOM-N and MBN all decreased with the increasing erosion depths. When considering on the restorative effects, manure amendments could eliminate the negative effect brought by soil erosion on POM-N, LFOM-N and MBN in erosion depths 5 cm and 10 cm.

**Table 1:** Labile organic nitrogen fractions from erosion depths 0 to 30 cm in two fertilization treatments.

Treatment	Erosion depth (cm)	MBN (mg kg^−1^)	POM-N (mg kg^−1^)	LFOM-N (mg kg^−1^)	DON (mg kg^−1^)
NPK	0	31.2 ± 2.0 c	570.5 ± 17.3 c	536.9 ± 18.5 c	16.7 ± 1.6 cde
	5	26.0 ± 3.7 cd	494.9 ± 12.8 d	492.7 ± 12.8 d	15.2 ± 1.3 def
	10	21.3 ± 2.0 de	445.3 ± 18.1 e	451.5 ± 17.0 e	13.9 ± 1.3 ef
	20	17.1 ± 1.8 e	409.1 ± 17.1 f	395.5 ± 12.0 f	12.1 ± 1.5 f
	30	16.9 ± 2.4 e	365.9 ± 16.8 g	351.1 ± 16.6 g	13.9 ± 2.0 ef
NPKM	0	43.8 ± 3.3 a	710.8 ± 10.1 a	611.5 ± 20.0 a	23.4 ± 2.3 a
	5	37.6 ± 2.6 b	657.1 ± 15.4 b	580.7 ± 13.9 b	22.5 ± 3.3 a
	10	29.9 ± 2.0 c	575.7 ± 13.0 c	535.1 ± 15.3 c	20.6 ± 2.0 ab
	20	22.7 ± 4.5 de	503.8 ± 18.2 d	457.2 ± 18.3 e	18.1 ± 2.2 bcd
	30	20.5 ± 6.2 de	435.2 ± 21.4 e	405.5 ± 24.3 f	19.8 ± 3.0 abc
**Source of variation (** ***p*** **values)**					
Treatment		<0.001	<0.001	<0.001	<0.001
Erosion depth		<0.001	<0.001	<0.001	<0.001
Treatment × Erosion depth		0.014	<0.001	0.032	0.047

NPK in the table represents the treatment with chemical fertilizers alone, and NPKM represents the treatment with chemical fertilizers plus manure amendment. Data were displayed as “mean ± SD”, n = 3. Different letters within columns indicate that treatment means were significantly different at the level of 0.05.

In contrast to POM-N, LOFM-N and MBN, DON did not change regularly. For erosion depths from 0 to 20 cm, DON declined but then increased at the erosion depth of 30 cm. For a specific erosion depth, DON was 40.12%~49.59% higher in the NPKM treatment than those in the NPK treatment.

### The proportions of labile organic nitrogen fractions in total nitrogen

**Figure 2:**
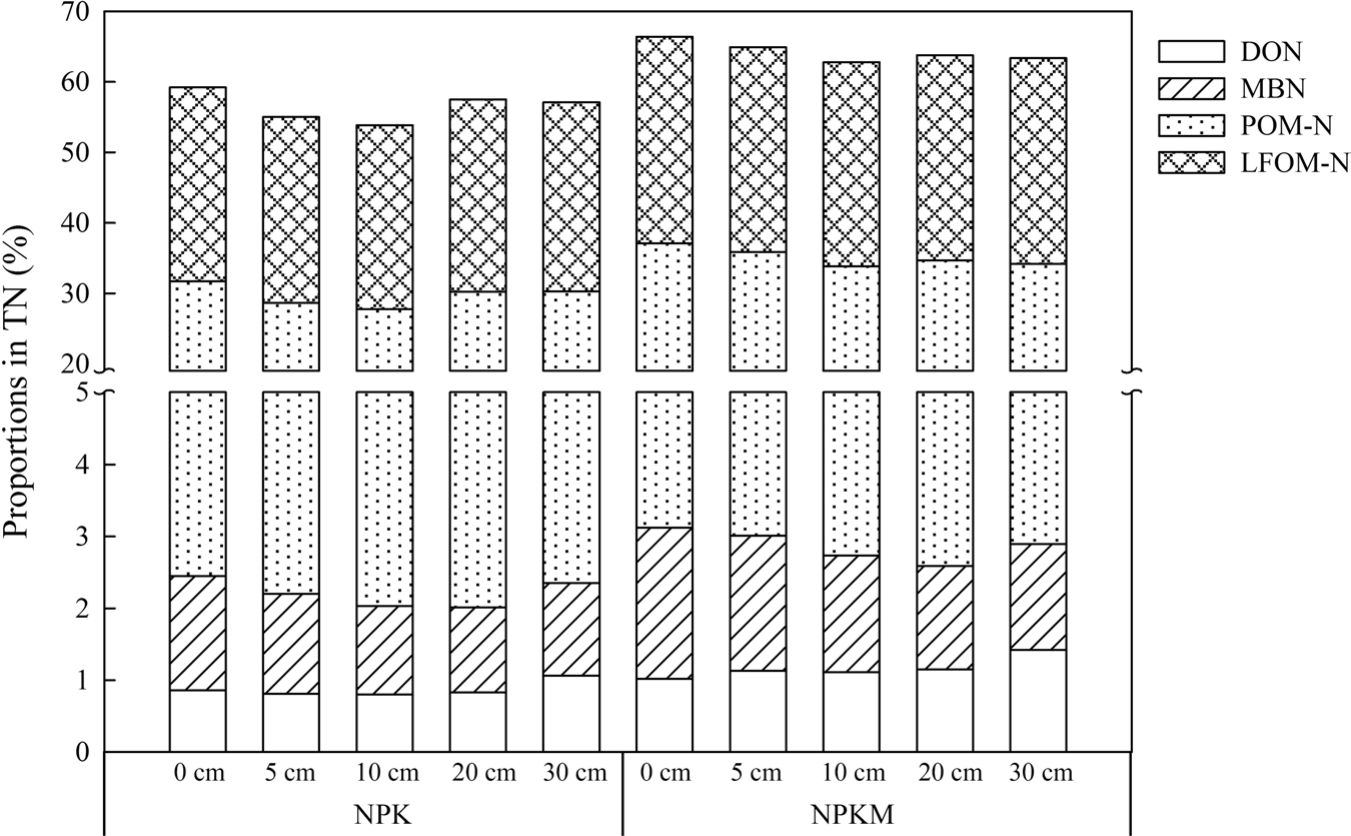
Proportions of labile organic nitrogen fractions in total nitrogen in NPK and NPKM treatments (%). NPK in the legends represents the treatment with chemical fertilizers alone, and NPKM represents the treatment with chemical fertilizers plus manure amendment.

Compared with the application of chemical fertilizer alone, 8-year manure amendment increased the proportions of POM-N, LOFM-N and MBN in TN. The proportions of both POM-N and LOFM-N in TN decreased with the increase in erosion depths from 0 to 10 cm; the proportions of POM-N and LOFM-N increased somewhat at the erosion depth of 20 cm but then decreased at the erosion depth of 30 cm. MBN decreased with the increase in erosion depth from 0 to 20 cm, but a small increase was observed at the erosion depth of 30 cm. The proportion of DON changed irregularly with erosion depth (Fig. 2). Within an erosion depth, the largest proportion of TN was POM-N and the smallest was that of DON; thus, POM-N contributed the most to TN, with DON contributing the least.

### Urease and protease activity and correlations with labile nitrogen fractions

**Figure 3:**
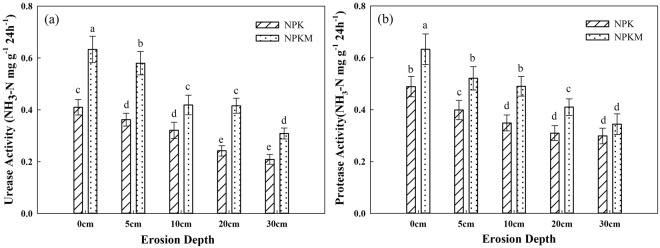
Urease activity (**a**) and protease activity (**b**) in Mollisols at different depths of erosion. NPK in the legends represents the treatment with chemical fertilizers alone, and NPKM represents the treatment with chemical fertilizers plus manure amendment. Different letters above bars indicate that means were significantly different at the level of 0.05. Bars represent standard errors (n = 3).

Eight-year manure amendment strongly affected urease and protease activity in eroded Mollisols (Fig. 3). Urease and protease activity both decreased with the increase in erosion depth. Manure amendment increased both urease and protease activity (*p* < *0*.*05*) compared with the treatment without manure.

Pearson’s correlations were conducted between enzyme activities and labile nitrogen fractions (Table 2). Urease activity was highly significantly positively correlated with POM-N, DON, MBN and LOFM-N (*p* < *0*.*01*). Protease activity was significantly positively correlated with POM-N, DON, MBN and LOFM-N (*p* < *0*.*05*).

**Table 2:** Correlation analyses between soil enzyme activities and labile organic nitrogen fractions.

Index	POM-N	LFOM-N	DON	MBN
Urease activity	0.95**	0.94**	0.94**	0.96**
Protease activity	0.87*	0.82*	0.79*	0.85*

Note: *Means significantly different at a level of 5%; **Means significantly different at a level of 1%.

## Discussion

The quantity of mineralized N derived from SON depends on microbial metabolism N concentrations contained in labile SON fractions^19^. In this study, application of manure increased POM-N, LFOM-N, DON and MBN in eroded Mollisols, which is similar to the effect of manure application on labile SON in other studies^41–44^. When the degree of erosion became more severe, the contribution of manure to POM-N, LFOM-N and MBN was lessened, but the contribution to DON showed no significant difference among erosion depths.

Decomposition of manure results in the formation of LFOM-N and POM-N, which are subsequently degraded by microorganisms for N supplying. Manure amendment enhanced POM-N and LOFM-N at all erosion depths. The increasing rates of POM-N dropped sharply when the degree of erosion became more severe, however the increasing rates of LFOM-N decreased slightly. This may be that POM-N has a lower C/N ratio relative to LFOM-N, which is more accessible to microorganisms^45^. When the erosion depth increased, less nutrients are available. To supply more N for both microorganisms and plant, the POM-N will be mineralized due to it is preferred by most microbes when compared to LFOM-N. Maybe more degradation caused the sharp drop of POM-N increasing rates and the less degradation contributed to the slight decrease in LFOM-N increasing rates.

The size of the MBN depends on C availability, the percentage of N and the C/N of organic amendments Manure amendment increases the soil microbial biomass^42^, thus resulting in an increase in MBN. However, with the increasing erosion depth, the contents of clay in the top layer also increased. More organic matter was binding to clay particles, which lower the microbial access^46,47^, thus resulting in a decrease in MBN and manure increasing rates in deep erosion depth. The decrease of MBN doesn’t mean a weak decomposition of labile SON fractions as MBN can only reflect the total amount of microbial biomass, but couldn’t represent the metabolic abilities of the microbiota. The metabolic abilities of the microbiota should be further studied.

Immediate and significant increases have been observed in soil dissolved organic matter content upon amendment with manure in many studies^18,48^, which results primarily from the presence of soluble materials in the amendments^49^. In this study, the large amount of DON in cattle manure caused a sharp increase in Mollisols and can conceal the difference in original soil DON. Another reason for the similarity of DON across all erosion depths may be leaching^50,51^ as DON can move with water in soil, considering our experiment was conducted on a slope with increasing erosion depth from the top to the bottom.

When considering on SNSC, the labile fractions should be selected for predicting it. POM-N is considered as the most important indicator for SNSC, as it is both a source of organic N that can be transformed into mineralized N and an accessible C source for microbes that can mineralize organic N from other sources^14^. LFOM-N is sometimes considered as an indicator for N immobilization, due to its relatively high C/N ratios^52^. However, in this study the C/N ratios of LFOM-N ranged from 7.7 to 18.5 (Table S1), which indicated that LFOM-N could also be also be used for N supplying due to that materials with a C:N ratio <25:1 will stimulate mineralization^1^. MBN and DON are regarded as transient, intermediary pools in soil^14,21^. Microbes can assimilate mineral N in soil and their residues can be a source for mineralization^9,10^. DON is generated as a by-product in the mineralization process^13,53^. DON may be used as a N source by soil microorganisms only under N limiting conditions^49^ and part of it may be mineralized to soil mineral N. In the present study, POM-N contributed 25.74–34.01% to TN and LFOM-N accounted for 26.10–27.26% of TN, which greatly exceeded the proportions of MBN and DON, so MBN and DON were excluded for predicting SNSC. Both POM-N and LFOM-N in this study were enhanced by manure amendment, indicating that manure amendment could enhance SNSC in eroded Mollisols.

Soil urease is strongly associated with soil nitrogen supplying ability and the activity can indicate the amounts of supplied nitrogen^54^. Protease transform proteins and peptides to amino acids, which is important for the nitrogen supply. Sui, *et al*.^39^ found that the activities of soil enzyme activities decreased as the erosion depth increased and concluded that when, insights into soil quality can be obtained from enzyme activities as enzyme activities were significantly correlated with soil nutrients. In present study, correlation analysis also showed that both urease and protease activities were significantly positively correlated with POM-N, DON, MBN and LOFM-N, respectively. Due to the complex composition of SON, labile SON fractions are both products and substrates of enzyme catalytic process^33^. More substrates would induce more enzymes and the enhanced enzyme activities would strengthen the catalysis of the biochemical process, thus producing more products^18^. The strong linkage between labile SON fractions and enzyme activities contributes most to the strong correlation. The strong correlation showed that urease and protease activity can indicate the overall change of labile N pools and worked similarly with labile SON fractions in predicting SNSC.

Manure amendment can improved SNSC and soil enzyme activities, even recover labile SON fractions in the light eroded Mollisols (erosion depth 5 cm and 10 cm) to normal level (erosion depth 0 cm in NPK treatment). The results provide more evidence for that manure application could recover eroded soils. Labile SON fractions in Mollisols with severe erosion (erosion depth 20 cm and 30 cm) couldn’t be fully restored by 8-year manure amendment. However, we do not recommend to apply manure continuously any more. First of all, 8-year amendment can recover the crop yield (Fig. S1)^40^. Second, the soil bulk density (Table S2) decrease sharply after 8-year continuously application of manure, which may result in plant lodging due to that crop roots won’t plunge deeply any more. Third, continuous application of manure may cause soil contamination, such as antibiotics pollution and heavy mental pollution.

## Conclusion

Eight-year application of manure significantly enhanced TN, labile SON fractions and enzyme activities in eroded Mollisols, which indicated that manure amendment was an effective way to improve SNSC. The strong positive correlation between labile SON fractions and enzyme activities also give an insight into predicting labile SON pools by using enzyme activity. However, eight-year manure amendment was not able to recover SNSC to the normal level, and other methods to restore eroded Mollisols need to be adapted to avoid negative effect brought by manure amendment.

## Materials and Methods

### Study site description

The field experiment was located at Guangrong Village, Hailun City, Heilongjiang Province, northeast China (47°21′N, 126°49′E). Mean annual temperature is 1.5 °C of which the effective accumulative temperature is 2200–2400 °C and mean annual precipitation is 500–600 mm of which 70% were received during June to September. Soil in this region was developed from glacial parent material with a 30 cm thick A horizon. The soil in this region was classified into Mollisol according to the soil texture classification system of the United States Department of Agriculture (USDA). Soybean was sowed in this area for more than 40 years. A soybean-corn rotation system was applied in this experiment from 2005 as corn became the popular crop in northeast China.

### Experimental design

To simulate different soil erosion levels in natural environment, 0, 5, 10, 20 and 30 cm of topsoil were removed in the fall 2004. The initial soil properties are listed in Table 3. Two treatments, namely chemical fertilizers alone (NPK) and chemical fertilizers plus manure amendment (NPKM), were implemented. In the NPK treatment, chemical fertilizers of 69 kg N ha^−1^, 69 kg P_2_O_5_ ha^−1^, and 7.5 kg K_2_O ha^−1^ were applied and mixed into the top 15 cm of soil before seeding. In the NPKM treatment, the application of chemical fertilizers was the same with NPK and 15,000 kg ha^−1^ on a dry weight basis of solid cattle manure were applied and mixed into the top 10 cm of soil after harvest the previous fall. Manure contained 730 g kg^−1^organic material, 20.7 g kg^−1^ total N, 7.9 g kg^−1^ total P, and 11.8 g kg^−1^ total K.A randomized complete block design was used with three replications. In total 30 plots were built, and each plot contained five rows, which was 5.0 m long by 0.7 m wide. The surrounding 0.7 m of each plot acts as guarding rows against different plots. The corn cultivar Haiyu 6 was sowed in early May each year. In early July, 69 kg N ha^−1^ was applied as topdressing in both treatments. More detailed experimental design can be seen in our previously publications^28,37,38,40^.

**Table 3:** Initial soil properties in 2004.

Erosion depth (cm)	SOC (g kg^−1^)	Total N (g kg^−1^)	Bulk density (g cm^−3^)	Field water holding capacity (%)	Saturation moisture capacity (%)	Soil particle distribution (%)		
						>0.02 mm	0.02–0.002 mm	<0.002 mm
0	31.36	1.81	1.02	23.2	43.34	34.13	30.85	35.02
5	30.49	1.77	1.14	25.01	50.56	36.14	28.3	35.56
10	28.92	1.68	1.12	23.15	44.47	36.36	26.41	37.23
20	24.05	1.4	1.12	23.09	49.29	34.69	27.24	38.07
30	22.39	1.3	1.17	24.56	55.48	33.67	27.5	38.83

### Soil sampling

Soil samples were collected in October 2012 after the corn harvest. The soil sample from each plot consisted of 15 sub-samples that were collected using a soil auger at depth 0–20 cm. After carefully removing the fine roots and other residues from the soil surface, each soil sample was divided into two parts. The first part was passed a 2 mm screen and stored at 4 °C for the analysis of MBN, DON, urease activity and protease activity. The second part was air-dried and then ground to pass a 2 mm mesh for the analysis of POM-N and LOFM-N and to pass a 0.149 mm mesh for the analysis of TN.

### Soil analysis

Approximately 25 mg soil was used for TN contents analysing by using an elemental analyzer (VarioEL III; Elementar, Germany).

MBN was determined by the chloroform fumigation-extraction method^55–57^ on soil samples stored at 4 °C. Each replicate was divided into three parts and the fresh soil weight in each part was equivalent to 25 g oven-dried soil. The first part was used to calculated soil water contents by oven-drying at 105 °C for 8 h. The second part was fumigated with ethanol-free chloroform for 24 h. The third part was the non-fumigation control. The second part and the third part had the same soil weight. Next, the soils were extracted using 50 mL 0.5 mol L^−1^ K_2_SO_4_, shaking at 180 rmin^−1^ for 30 min. The organic N was analysed using an automated TOC Analyzer (Elemental Liquid TOC II, Elementar, Germany). MBN was calculated by the following equation:

$${\rm{MBN}}=({{\rm{E}}}_{{\rm{N}}}/{{\rm{K}}}_{{\rm{N}}})/{\rm{W}}$$

where E_N_ is the difference which the organic C in fumigated soil minus that in the non-fumigated soil, K_N_ = 0.45 and W is the oven-dried weight.

DON was determined by modified method of Ge, *et al*.^58^. Briefly, fresh soil samples equivalent to 3 g oven-dried weight were extracted with 30 mL distilled water in a 50 mL polypropylene centrifuge tubes. The tubes were shaken for 30 min at 300 r min^−1^ and at 20 °C. Then the mixture was centrifuged at 4500 r min^−1^ for 15 min before filtering through 0.45 μm filters. The filtrate was analysed using an automated TOC Analyzer (Elemental Liquid TOC II, Elementar, Germany).

After adding 30 mL 5 g L^−1^ (NaPO_3_)_6_ 10 g air-dried soil, the mixture was shaken for 18 h at 180 r min^−1^ and at 20 °C to separate POM-N. The separated soil was passed through a 53 μm screen. All material remaining on the mesh was oven-dried at 50 °C for 18 h^59,60^. Then the N contents in particle organic matter were determined using the TN method, which has been described above.

To separate LFOM-N, 30 mL 1.7 g cm^−3^ NaI was added into a 50 mL polypropylene centrifuge tube containing15 g air-dried soil After shaking 1 h at 180 r min^−1^, the tubes were centrifuged at 4500 r min^−1^ for 15 min. Then pouring the supernatant into filter to collect the components of low density (<1.7 g cm^−3^). To transfer the remains on the filter paper to a dish, 0.05 mol L^−1^ CaCl_2_ and distilled water were used. The dish was oven-dried at 50 °C for 48 h^61,62^. The dried material was ground to pass a 0.149 mm mesh for N contents analysis by the TN method.

Estimation of urease activity was carried out by indophenol-blue colorimetry and protease activity was determined by ninhydrin colorimetry^63^.

### Data Analysis

Data was collected and assessed using IBM SPSS 20.0. In all tables and figures, data was displayed as means. A two-way ANOVA procedure was adapted to analyse TN, labile organic nitrogen fractions and enzyme activities, using fertilization and erosion depth as fixed factors. The difference between fertilization treatments among erosion depths was compared using Duncan’s test and the significant level is α = 0.05. A Pearson’s correlation analysis was performed to assess the relationship of enzyme activities with labile organic N fractions.

## Electronic supplementary material

Supplementary Materials

## Data Availability

All data generated or analysed during this study are included in this published article. For the original data, they are available from the corresponding author on reasonable request.
