# Supplementary material for: Responses of Labile Organic Nitrogen Fractions and Enzyme Activities in eroded Mollisols After 8-year Manure Amendment
Source: Sci Rep. 2018 Sep 21;8:14179. doi: 10.1038/s41598-018-32649-y (PMC6155099; doi:10.1038/s41598-018-32649-y)
Supplement: Supplementary file 1 — Supplementary Materials [file 41598_2018_32649_MOESM1_ESM.docx]

Responses of Labile Organic Nitrogen Fractions and Enzyme Activities in eroded Mollisols After 8-year Manure Amendment

Yi-min Chen^1, 2, +^, Xin Xu^3, +^, Xiao-guang Jiao^3*^, Yue-yu Sui^1*^, Xiao-bing Liu^1^, Jin-yuan Zhang^1, 2^, Ke Zhou^3^, Jiu-ming Zhang^4^

1. Key Laboratory of Mollisols Agroecology, Northeast Institute of Geography and Agroecology, Chinese Academy of Sciences, 150081, Harbin, China
2. University of Chinese Academy of Sciences, 100049, Beijing, China
3. College of Agricultural Resources and Environment, Heilongjiang University, 150080, Harbin, China
4. Institute of Soil and Fertilizer and Environment Resources, Heilongjiang Academy of Agricultural Sciences, Harbin 150086, China

* Corresponding authors: Yue-yu Sui, E-mail: [suiyy@iga.ac.cn](mailto:suiyy@iga.ac.cn)

Xiao-guang Jiao, E-mail: 2004086@hlju.edu.cn

+ These authors contributed equally to this work


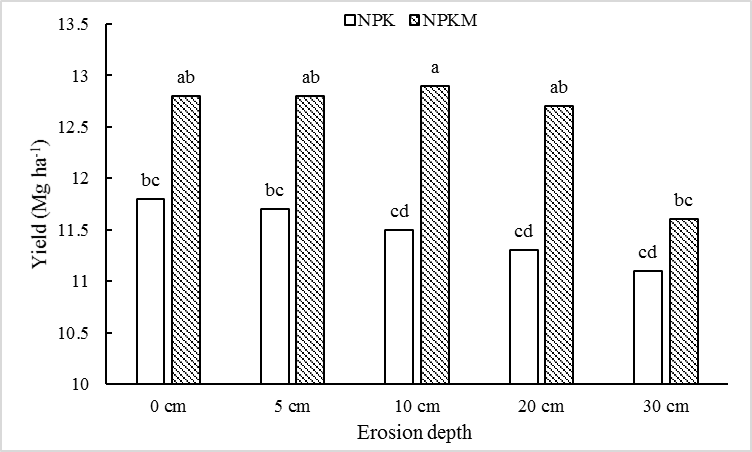


**Fig. S1** Maize grain yields in NPK and NPKM. NPK in the legend represents the treatment with chemical fertilizers alone, and NPKM represents the treatment with chemical fertilizers plus manure amendment. Different letters above bars indicate that treatment means were significantly different at the level of 0.05.

**Table S1** C/N ratios of labile organic matter fractions.

| Treatment | Erosion depth  (cm) | MBC/MBN | POM-C/POM-N | LFOM-C/LFOM-N | DOC/DON |
| --- | --- | --- | --- | --- | --- |
| NPK | 0 | 5.8 | 14.0 | 15.1 | 1.0 |
|  | 5 | 6.2 | 13.8 | 14.5 | 1.0 |
|  | 10 | 6.5 | 13.1 | 13.6 | 0.8 |
|  | 20 | 7.1 | 10.2 | 10.7 | 1.1 |
|  | 30 | 6.6 | 6.5 | 7.7 | 0.9 |
| NPKM | 0 | 6.2 | 15.4 | 18.1 | 0.9 |
|  | 5 | 5.9 | 15.1 | 17.9 | 0.9 |
|  | 10 | 6.7 | 14.9 | 18.5 | 1.0 |
|  | 20 | 7.6 | 14.2 | 17.7 | 1.2 |
|  | 30 | 7.6 | 10.4 | 15.8 | 0.8 |

**Table S2** Soil bulk densities in NPK and NPKM.

| Treatment | Erosion depth  (cm) | Bulk density  （g cm^-3^） |
| --- | --- | --- |
| NPK | 0 | 1.10 |
|  | 5 | 1.13 |
|  | 10 | 1.15 |
|  | 20 | 1.16 |
|  | 30 | 1.18 |
| NPKM | 0 | 0.92 |
|  | 5 | 0.94 |
|  | 10 | 0.97 |
|  | 20 | 0.97 |
|  | 30 | 0.99 |
